# A Rare Cause for Acute Chest Pain in the Emergency Setting That Is Hard to Swallow

**DOI:** 10.1155/2013/646342

**Published:** 2013-07-17

**Authors:** Eric Cummins, Meenal Sharkey, Travis Eastin, Eric Adkins

**Affiliations:** Department of Emergency Medicine, The Ohio State University Medical Center, 750 Prior Hall, 376 W 10th Avenue, Columbus, OH 43210, USA

## Abstract

Intramural esophageal hematoma is a very rare but important cause of chest pain. This condition shares similarity with the diagnosis of other thoracic emergencies and has a high potential for misdiagnosis. The emergency clinician plays a critical role in the early identification and management of these patients. The management of intramural hematomas is typically conservative, and a misdiagnosis could lead to deleterious effects. Preexisting coagulopathy is one of the major risk factors. With the advent of new anticoagulation medications to prevent thromboembolic events, it is important that emergency medicine providers expand the differential diagnosis of chest pain.

## 1. Introduction

An intramural esophageal hematoma (IEH), also described as incomplete Boerhaave's syndrome, intramural dissection, or intramural hemorrhage, is a rare condition. The presenting symptoms are nonspecific but may include chest pain, back pain, dysphagia, hematemesis, or globus hystericus. Because it can be associated with esophageal perforation, IEH should be considered in emergency department patients with retrosternal chest pain, dysphagia, hematemesis, and odynophagia [1]. We present a case of substernal chest pain in a patient anticoagulated with dabigatran who was initially diagnosed with a distal esophageal impaction but instead had this rare condition. Lastly, we perform an in-depth review of the literature and case reports regarding evaluation and management of intramural esophageal hematomas.

## 2. Case Presentation

An 81-year-old female with a past medical history significant for atrial fibrillation status post recent cardiac ablation on dabigatran (Pradaxa, Boehringer Ingelheim Pharm) presented to a local emergency department (ED) for evaluation of chest pain that started immediately after drinking juice. The pain was described as similar to previous episodes, but when the pain did not remit, she went to her local ED. An electrocardiogram (EKG) and laboratory studies were obtained, which were significant for normal sinus rhythm without ST changes and a hemoglobin of 13.9 g/dL (8.63 mmol/L; range: 12.0–16.0 g/dL; 7.4–9.9 mmol/L) [2]. Due to concern for an aortic aneurysm, computed tomography angiography (CTA) was performed which demonstrated a distal impaction of her esophagus, with greatest suspicion for impacted food bolus.

The patient was transported to our tertiary care ED for a gastroenterology consult and endoscopy. 

On arrival to the tertiary care ED, the patient was hemodynamically stable. Cardiac, respiratory, and abdominal examinations were unremarkable. Initial evaluation included an EKG and basic laboratory studies, which were obtained approximately eight hours after her initial laboratory studies. The EKG showed a normal sinus rhythm without ST segment elevations. Laboratory studies were significant for a hemoglobin 10.1 g/dL (6.27 mmol/L), prothrombin time 19.5 seconds (reference range 11.1–13.1 seconds) international normalized ratio 1.6, and white blood cell count 11,300/mm^3^ (11.3 × 10^9^/liter; reference range: 4,400–11,000/mm^3^, 4.5–11 × 10^9^/liter). Her platelet count, troponin, and liver enzymes were within normal limits. A chest X-ray did not reveal an acute abnormality. After failure to respond to glucagon, the GI service was consulted.

**Figure 1:**
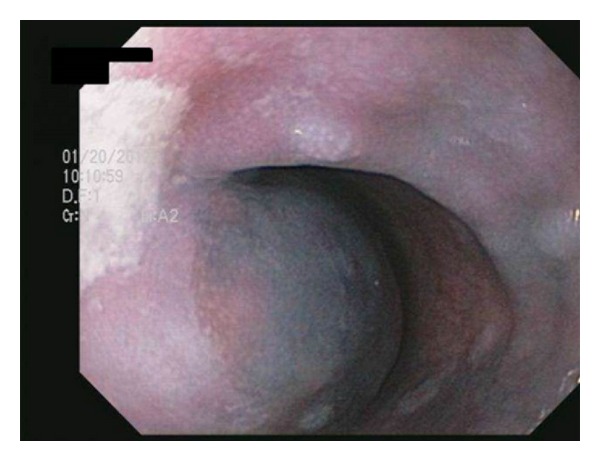
Upper endoscopy of the middle third of the esophagus demonstrating extrinsic compression (bulging) into the lumen.

**Figure 2:**
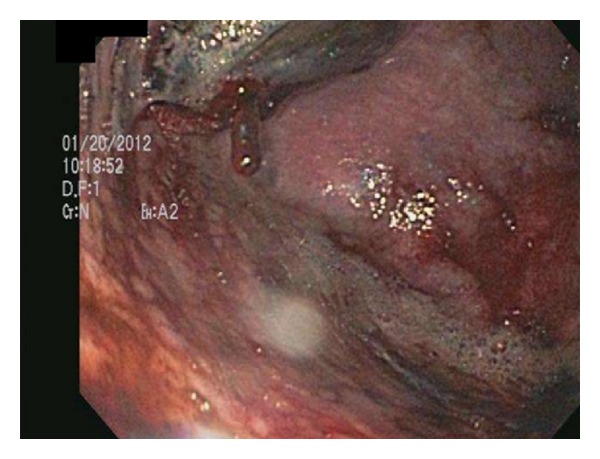
Upper endoscopy of the distal third of the esophagus/cardia revealing the hematoma. Notice the slow oozing of blood from the cardia aspect.

**Figure 3:**
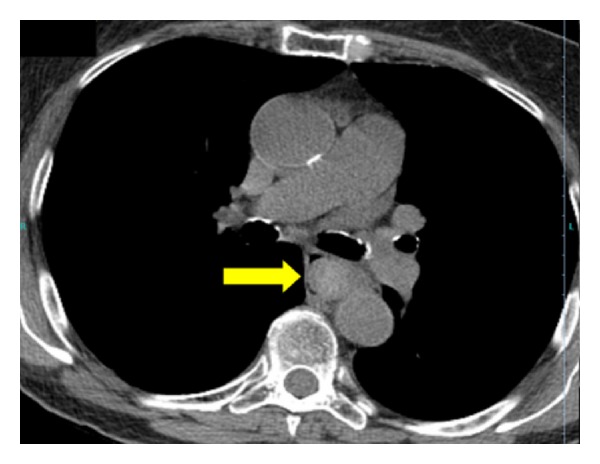
CT of middle esophagus revealing a large intraluminal hematoma obstructing the lumen. Notice that there is only a thin layer of air between the hematoma and esophageal wall (arrow).

**Figure 4:**
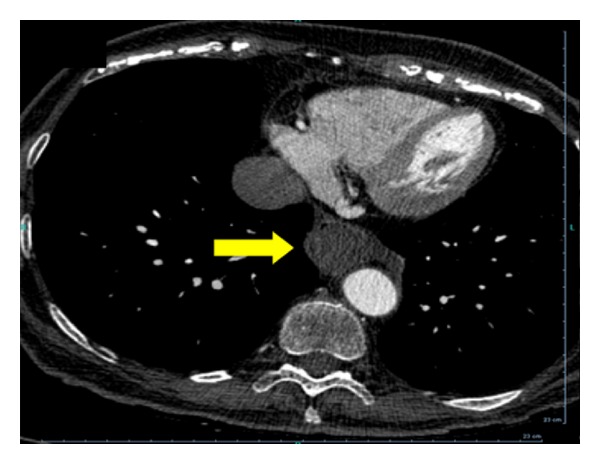
CT of distal esophagus revealing near-complete occlusion of the lumen (arrow).

**Figure 5:**
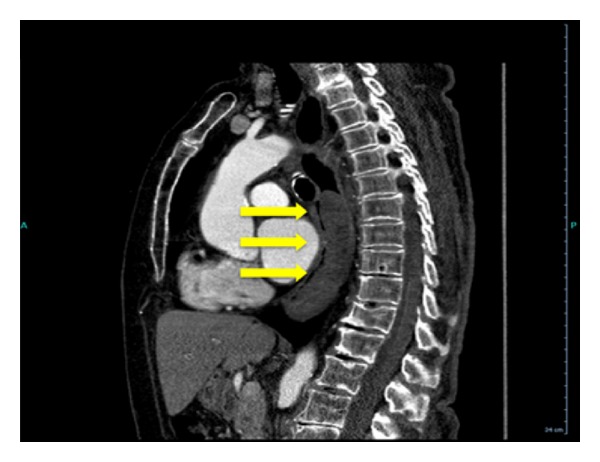
CT sagittal view of esophageal hematoma (arrows).

Upper endoscopy was performed, and the patient was found to have extrinsic compression of the middle and lower third of her esophagus (Figures 1 and 2). The patient was transported back to the ED for computed tomography (CT) of the chest (Figures 3, 4, and 5) and evaluation by the thoracic surgery team. The CT scan revealed a large intramural esophageal hematoma, measuring 2.4 cm × 2.6 cm and spanned a length of 13 cm craniocaudally. There was no evidence of mediastinal air, perforation, or pericardial effusion. The dabigatran was discontinued upon initial diagnosis of esophageal hematoma. 

**Figure 6:**
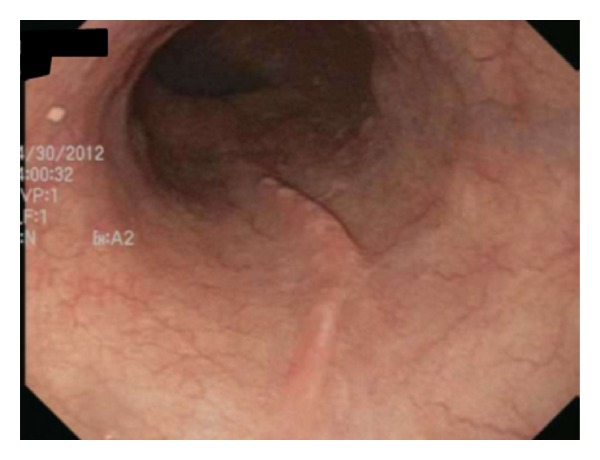
Upper endoscopy of the distal third of the esophagus showing full resolution of hematoma. EGD performed 3 months after presentation.

The patient was admitted to the hospital for observation and serial hemoglobin levels. She did not require transfusion. The thoracic surgery team determined that surgical intervention was not required. The cardiac electrophysiology (EP) service evaluated the patient while in the hospital. Given the risk of an expanding hematoma with continued anticoagulation, antiarrhythmic therapy and low-dose aspirin were initiated for management of the patient's atrial fibrillation. She was discharged home in stable condition after an uncomplicated hospital stay of 4 days with recommendation to continue a soft diet. An EGD performed 3 months later showed full resolution of the IEH (Figure 6).

## 3. Discussion

Since the first documented case by Marks and Keet in 1968 [3], intramural esophageal hematomas have been a rare but potentially serious cause of chest pain. The pathophysiology of IEH is hemorrhage that dissects the submucosal tissue layers [4]. Typical clinical manifestations include retrosternal, nonpleuritic chest pain; odynophagia; hematemesis, and dysphagia. The classic triad of chest pain, dysphagia, and hematemesis is present in only 35% of cases [5]. Unfortunately, physical examination is often limited and unreliable in the setting of IEH.

Although intramural esophageal hematoma is a rare entity, it is important for the emergency medicine physician to discriminate it from other thoracic emergencies such as acute coronary syndrome, thoracic aortic dissection, pulmonary embolism, perforated peptic ulcer, and Boerhaave's syndrome. This condition shares similarity with the diagnosis of myocardial infarction and not infrequently is deleteriously treated with thrombolytic therapy [6]. 

The most common location for IEH, reported in 83% of cases, is the distal third of the esophagus, as this location is devoid of striated muscle and has the least support from surrounding structures (i.e., heart and trachea) [7, 8]. 

IEH has been reported secondary to endotracheal intubation, foreign body ingestion, pill-induced esophageal injury, vomiting, coagulopathy, and endoscopic procedures [9, 10]. Spontaneous IEH is typically associated with women of middle or advanced age [11]. In our patient, there was concern that the IEH may have been a complication of the recent cardiac ablation. 

In a review of the literature, we found only two reports of esophageal hematomas complicating cardiac catheter ablation procedures. McCall and Thomas felt that the esophageal hematoma likely occurred due to postanesthesia emesis during the recovery period in the setting of anticoagulation and was unlikely caused by unintentional thermal injury [12]. The second case, presented by Nguyen and colleagues, concluded that the esophageal hematoma was a result of local trauma from the preprocedural transesophageal echocardiogram and was exacerbated by anticoagulation therapy [13]. Of note, the more serious and more common esophageal complication from cardiac ablation is atrioesophageal fistulas which is thought to be a direct result from thermal injury [14]. 

The diagnosis of IEH can be made by direct visualization with upper endoscopy and various imaging modalities. With endoscopy, IEH often appears as submucosal, smooth, raised purple-red lesions that protrude into and occlude the lumen [15]. Previously, a contrast swallow study was used to assess for an intraluminal filling defect in the esophagus and compression of the lumen by the hematoma; however, with increasing advancements in multislice CT technology, the CT scan has supplanted contrast swallow studies as the best initial imaging study [16]. Computed tomography of the chest often demonstrates luminal wall thickening of the esophagus, with density similar to that of blood [4]. A CT scan is advised in most cases to determine the extent of the hematoma, evaluate for atrioesophageal fistula, assess for perforation/extravasation, and determine the age of the hematoma through measurement of the attenuation values within the lesion [8, 14]. Occasionally, a “double barrel” sign can be seen, representing a false lumen inside the esophageal wall [17]. When visualized on CT, IEH can be misinterpreted as malignancy, or as in our patient, as an esophageal food impaction. Endoscopic ultrasound or MRI may be beneficial in establishing the diagnosis [14, 17, 18]. 

Esophageal hematomas generally follow a benign clinical course. Kerr demonstrated that supportive care alone for esophageal hematomas leads to full resolution in 80% of patients [19]. An appropriate therapy in the emergency setting is supportive and includes nil per os (NPO), intravenous fluid hydration, analgesia, and correcting any underlying coagulopathy [10]. There have been case reports of IEH associated with perforation and mediastinitis requiring immediate surgical intervention [20]. In a case series of 4 patients, Shay and colleagues revealed that no deaths occurred from immediate complications of esophageal hematomas [21]. In that series, only one patient died as a complication from an esophagectomy 6 months later.

## 4. Conclusion

Spontaneous intramural esophageal hematoma is a rare disease entity that may occur more commonly in women of middle age and older who present with odynophagia and retrosternal, nonpleuritic chest pain. The relative contribution of anticoagulation, recent transesophageal echocardiogram, and cardiac ablation in this patient is unknown but likely contributory. Morbidity and mortality are low if identified in a timely fashion and treated with supportive care and discontinuation of anticoagulation.
